# A Commercial Potential Blue Pea (*Clitoria ternatea* L.) Flower Extract Incorporated Beverage Having Functional Properties

**DOI:** 10.1155/2019/2916914

**Published:** 2019-05-20

**Authors:** Suraweera Arachchilage Tharindu Lakshan, Nileththi Yasendra Jayanath, Walimuni Prabhashini Kaushalya Mendis Abeysekera, Walimuni Kanchana Subhashini Mendis Abeysekera

**Affiliations:** ^1^Department of Food Science & Technology, Faculty of Agriculture, University of Peradeniya, Peradeniya 20400, Sri Lanka; ^2^Herbal Technology Section, Industrial Technology Institute, Malabe, Sri Lanka; ^3^Department of Agricultural Technology, Faculty of Technology, University of Colombo, Sri Lanka

## Abstract

*Clitoria ternatea *L. commonly known as ‘blue pea' is an underutilized plant in Sri Lanka. The blue coloured flower of this plant is used in medicine in Sri Lankan traditional medical system and also reported to have several health benefits in recent findings at the international level. However, to date scientifically validated value added products from blue pea flower (BPF) is very limited worldwide. In this connection, this study was carried out to develop a commercial potential blue pea flower extract (BFE) incorporated beverage having functional properties. Dried BPFs were extracted into water with varying flower: water ratio, temperature, and time using response surface methodology (RSM) along with Box–Behnken design. A range of BFE incorporated beverages was developed comprising a natural sweetener (Stevia extract) and a flavour (lime). The most acceptable formulation was selected via ranking and hedonic sensory tests. Further, it was evaluated for functional properties in terms of antioxidant activity via total polyphenolic and flavonoid contents, ferric reducing antioxidant power and radical scavenging activities via ORAC; DPPH and ABTS^. ^Glycaemic regulatory properties (GCP) were evaluated in terms of antiamylase and antiglucosidase activities. Quality parameters of the developed beverage were evaluated for a period of 28 days at different time intervals and a colour chart was also developed. The optimum conditions for extraction of BPF via RSM were 3 g of powdered BPF/L of water at 59.6 °C for 37 min. The most acceptable formulation consists of BFE, Stevia extract, and lime at a ratio of 983.25:1.75:15. Further, it had significantly higher (p<0.05) consumer preference for sensory attributes. Further, it possesses an antioxidant activity through multiple mechanisms while GCP were not detected. Moreover, it was shelf stable for a period of 28 days without preservatives. The colour chart can be used to monitor the quality of the beverage.

## 1. Introduction

*Clitoria ternatea *L., commonly known as ‘blue pea' [1], is a perennial twinning herbaceous plant which belongs to the family Fabaceae. The plant is mainly distributed in the tropical regions of India, Sri Lanka, Malaysia, Burma, and Philippine islands [2, 3]. It has two main varieties based on the colour of the petals, namely, white and blue flowered varieties. Different parts of this plant have been used in Sri Lankan traditional system of medicine and in folklore to treat variety of disorders such as anasarca, ascites, liver problems, hemicrania, irritation of urethra and bladder, and enlargement of abdominal viscera [4]. Further, the medicinal properties of this plant are scientifically validated particularly at international level and reported to have several biological activities such as antioxidant, antidiabetic, and hepatoprotective properties [5, 6].

Plants with medicinal properties are good alternative sources to find remedies for existing noncommunicable diseases worldwide [7]. Further, numerous studies showed that foods rich in antioxidants play a pivotal role in prevention and management of range of oxidative stress associated chronic diseases. The mechanisms of antioxidants in managing oxidative stress in biological systems are diverse which included scavenging of free radicals, inhibition of oxidative enzymes, chelation of metal ions, and acting as antioxidant enzyme cofactors [8, 9]. Therefore, diets rich in antioxidants could be a better alternative source to manage diabetes mellitus, since oxidative stress plays a major role in the development of diabetes mellitus and its complications.

According to the recent statistics, there were 425 million people with diabetes in year 2017 worldwide and the number is predicted to be 629 million in year 2045 [10]. Although antidiabetic drugs and insulin regimes are very effective in managing diabetes mellitus still there is no permanent cure for this disease [11]. Therefore, the search of novel drug leads/functional foods from natural sources preferably from medicinal plants with no/minimum side effects is timely important. As such alpha-amylase and alpha-glucosidase inhibitors are key targets since these two enzymes play a key role in the digestion of carbohydrates. Therefore, inhibition of these enzymes is reported to have antidiabetic activity [12]. Further, there are several findings highlighting that antioxidants such as polyphenolics involve in inhibition of alpha-amylase and alpha-glucosidase enzymes [13].

*Clitoria ternatea *L., the ‘blue pea', is an underutilized plant in Sri Lanka. Although the medicinal property of this plant is well documented in traditional medicine, it is not properly harnessed to date. Indeed, there are no scientific reports on the health benefits of this plant including flowers and there are no value added products in Sri Lanka. Currently, there are few products of flowers of* Clitoria ternatea *L. in the world market. However, to the best of our knowledge, there are very limited scientifically validated value added products of flowers of* Clitoria ternatea *L. even in the world. In this connection, this study was carried out to develop a commercial potential* Clitoria ternatea *L. flower extract incorporated beverage having functional properties.

## 2. Materials and Methods

### 2.1. Sample Collection

Blue coloured, fully bloomed, disease free, and undamaged healthy flowers of* Clitoria ternatea* L. (blue flower) were collected from home gardens of Athurugiriya, Colombo, Sri Lanka. Stevia syrup (90% w/v) was purchased from RCC Global (M) SDN BHD, Johor, Malaysia. Fully ripened lime fruits were purchased from the retail shops in Peradeniya, Sri Lanka.

### 2.2. Sample Preparation

Diseased free, undamaged blue pea flowers were oven dried (Model SPF-600, SIBATA, Japan) at 50°C for 24 h [14]. Dried flowers were ground using a domestic grinder (Model MX-AC400, Panasonic, India) for 5 min, sieved (1 mm sieve) and kept in sealed airtight low-density polyethylene bags (300 gauge) at room temperature until use for the analysis. Lime juice was extracted immediately before (within 10 minutes) adding to the herbal beverage, using a domestic squeezer (Model EN1031, Evernew, China), followed by filtering using a clean muslin cloth.

### 2.3. Optimization of Extraction Procedure of Blue Pea Flower

Powdered blue pea flowers were extracted into water (Model: D-91126 Schwabach FRG, Memmert, Germany) with varying temperature (A), time (B), and flower: water (F: W) ratio (C) as given in Table 1. Extracts were filtered (0.45 *μ*m, 25 mm filter) and total polyphenolic content (TPC) was determined by 96 well microplate based Folin-Ciocalteu method [15]. Total phenolic content was expressed as mg gallic acid equivalents (GAE) per litre of extract.

The effect of extraction conditions (A, B, and C) on the response variable, TPC, was evaluated by Box–Behnken design (Table 1), along with the Design Expert Software (Version 10.0.0, Stat-Ease Inc, Minneapolis, MN, USA). The experimental design consisted of 15 treatment combinations including 3 center points and run in a randomized order to reduce unexpected variations (n=3 for each experimental point). Data was fitted into the following quadratic polynomial regression model:

(1) $$\begin{array}{c} Y=\beta_{0}+Ʃ\beta_{i}X_{i}+Ʃ\beta_{ii}X_{i2}+Ʃ\beta_{ij}X_{i}X_{j} \end{array}$$

where Y is the response variable, *β*_0_ represents the constant, *β*_i_, *β*_ii_, and *β*_ij_ represent linear, quadratic, and interactive coefficients, respectively, identified by the model, and X_i_ and X_j_ are the independent variables. Desirability function was used to determine the optimized conditions (A, B, and C) of extraction procedure of blue pea flower in order to get a maximum TPC.

### 2.4. Development of a Functional Beverage

**Figure 1:**
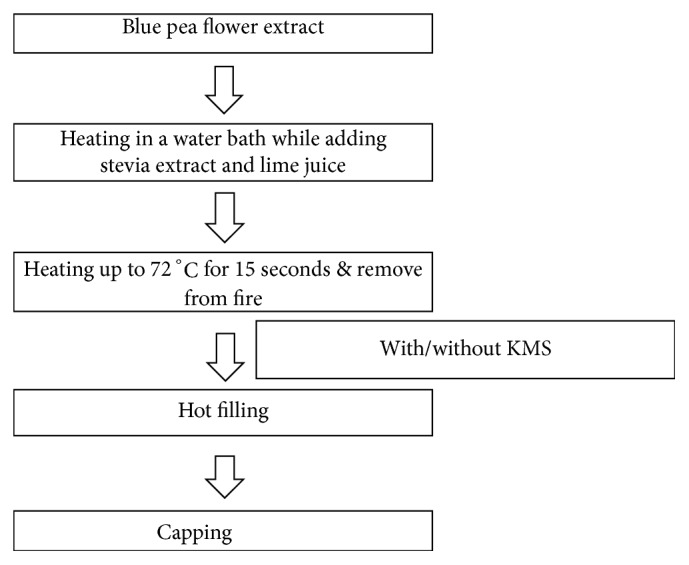
Process flow diagram of preparation of blue pea flower extract incorporated beverage.

Optimum treatment condition is where 3 g of powdered blue pea flowers in 1 L of distilled water at 59.6°C for 37 minutes were used for the extraction. The strained extract was processed into the beverage as given in Figure 1. Two sets of beverages were developed with and without adding potassium metabisulphite (KMS) while keeping other parameters constant. Initially, different experiments were conducted to identify acceptable levels of lime juice (15, 20, 25 g/L) and extract of stevia (1.50, 1.75, 2.00 mL/L). Finally, 9 formulations were developed and 3 formulations were selected for further analysis based on the preference to different sensory attributes. The selected 3 formulations were subjected to a ranking test based on the preference of colour, aroma, lime flavour, sweetness, and overall acceptability of the beverage, using 41 semitrained panellists (male: female ratio - 9:11). In the ranking test, formulations were rated from 1 to 3 based on the preference of panellists for the tested sensory attributes where most preferred formulation rated as 1 and least preferred formulation rated as 3. The most preferred formulation was further subjected to a hedonic test (9-point scale: 1- dislike extremely, 2- dislike very much, 3- dislike moderately, 4- dislike slightly, 5- neither like nor dislike, 6- like slightly, 7- like moderately, 8- like very much, and 9- like extremely) to evaluate the consumer acceptability for colour, aroma, lime flavour, sweetness, and overall acceptability using 41 semitrained panellists (male: female ratio- 2:3). In both sensory tests, the panellists used had a good grasp on sensory evaluation techniques and the rating scales. Further, the panellists were well guided for carrying out the tests by prediscussion sessions and structured instructions in the sensory ballot. All the sensory tests were conducted at the sensory laboratory of the Department of Food Science & Technology, Faculty of Agriculture, University of Peradeniya, Sri Lanka.

### 2.5. Chemicals and Reagents

Gallic acid, Folin-Ciocalteu phenol reagent, 6-hydroxy-2,5,7,8-tetramethylchroman-2 carboxylic acid (Trolox), 2,2'-Azino-bis (3-ethylbenzothiazoline-6-sulfonic acid) diammonium salt (ABTS), 1,1-diphenyl-2-picrylhydrazine (DPPH), 2, 2'-azobis (2-amidino-propane) dihydrochloride (AAPH), 2,4,6-tripyridyl-s-triazine (TPTZ), potassium persulfate, ferric chloride, fluorescein, p-nitrophenyl-*α*-D-glucopyranoside (PNPG), sodium acetate, DNS reagent, acarbose, sodium carbonate and alpha-glucosidase (type V from rice) enzyme were purchased from Sigma-Aldrich Inc., USA. alpha-amylase (*Bacillus amyloliquefaciens*) enzyme was purchased from Roche Diagnostics, USA. All other chemicals used were of analytical grade.

### 2.6. Evaluation of Antioxidant and Antidiabetic Activities of the Blue Pea Flower Extract and Blue Pea Flower Extract Incorporated Functional Beverage

#### 2.6.1. Evaluation of Antioxidant Activity

(*1) Total Phenolic Content (TPC)*. Total phenolic content of blue pea flower extract (BFE), blue pea flower extract incorporated functional beverage (BFD), and control experiment replacing blue pea flower extract with distilled water (BFC) were determined by Folin-Ciocalteu method as described by Singleton* et al*. (1999) [15] with minor modifications. Twenty microliters of samples were added to 110 *μ*L of freshly prepared (10 times diluted) Folin-Ciocalteu reagent followed by addition of 70 *μ*L of sodium carbonate solution to the mixture. Then mixture was incubated at room temperature (25±2 °C) for 30 minutes and absorbance was measured at 765 nm using a microplate reader (SpectraMax Plus, Molecular Device, USA). Gallic acid was used as the standard (assay concentrations: 3, 6, 12, 25, 50, and 100 *μ*g/mL). Results were expressed as mg gallic acid equivalents (GAE) per litre.

(*2) Total Flavonoid Content (TFC)*. Aluminium chloride method was used to determine the total flavonoid content of BFE, BFD, and BFC as described by Siddhuraju and Becker (2003) [16] with slight modification. One hundred microliters of 2% methanolic aluminium chloride solution was added to 100 *μ*L of BFE, BFD, and BFC (assay concentration: 125 *μ*L/mL) and the mixture was incubated at room temperature (25±2 °C) for 10 minutes. The absorbance was recorded using a microplate reader (SpectraMax Plus, Molecular Device, USA) at 415 nm. Quercetin was used as the standard (assay concentrations: 0.49, 0.98, 1.96, 3.91, 7.82, 15.63, 31.25, and 62.50 *μ*g/mL). Results were expressed as mg quercetin equivalents (QE) per litre.

(*3) Ferric Reducing Antioxidant Power (FRAP)*. Ferric reducing antioxidant power of BFE, BFD, and BFC were determined according to the method of Benzie and Szeto (1999) [17] with some modifications in 96 well microplates. Ten millimolar TPTZ solution was prepared in 40 mM HCl. Three hundred millimolar acetate buffer (pH 3.6), 10 mM TPTZ solution, and 20 mM FeCl_3_.6H_2_O were mixed in a ratio of 10:1:1 to prepare the working FRAP reagent just before use. The reagent was incubated at 37 °C for 10 minutes. A reaction volume of 200 *μ*L containing 30 *μ*L of acetate buffer, 150 *μ*L of working FRAP reagent, and 20 *μ*L of sample was incubated at room temperature (30±2 °C) for 8 minutes and absorbance was recorded at 600 nm using a microplate reader (SpectrMax Plus, Molecular Device, USA). Trolox was used as the standard (assay concentrations: 0.49, 0.98, 1.96, 3.91, 7.82, 15.63, 31.25, and 62.50 *μ*g/mL). The results were expressed as mg Trolox equivalents (TE) per litre.

(*4) DPPH Radical Scavenging Activity*. Determination of DPPH radical scavenging activity of BFE, BFD, and BFC was carried out by the method of Blois (1958) [18]. A reaction volume of 200 *μ*L containing 125 *μ*M DPPH radical and 50 *μ*L of different concentrations of BFE, BFD, and BFC (assay concentrations: 62.50, 31.25, 15.63, and 7.81 *μ*L/mL in methanol) was incubated at 25±2 °C for 15 minutes. After the incubation period absorbance was recorded at 517 nm using a microplate reader (SpectrMax Plus, Molecular Device, USA). Trolox was used as the standard antioxidant (0.78, 1.56, 3.125, 6.25 and 12.5 *μ*g/mL). Results were expressed as mg Trolox equivalents (TE) per litre. IC_50_ value was expressed as *μ*L of extract/1 mL of reaction volume.

(*5) ABTS*^+^* Radical Cation Scavenging Activity*. The ABTS+ radical scavenging activity of BFE, BFD, and BFC was determined according to the method described by Re* et al.* (1999) [19]. A reaction volume of 200 *μ*L containing 40 *μ*M ABTS^+^ radical and 50 *μ*L of different concentrations of BFE, BFD, or BFC (assay concentrations: 62.50, 31.25, 15.63, 7.81, 3.91, 1.95, 0.98, 0.49 and 0.24 *μ*L/mL) was incubated at 25±2 °C for 10 minutes. After the incubation period, absorbance was recorded at 734 nm using a microplate reader (SpectrMax Plus, Molecular Device, USA). Trolox was used as the standard antioxidant (25.00, 12.50, 6.25, 3.12, and 1.56 *μ*g/mL). Results were expressed as mg TE per litre of sample. IC_50_ value was expressed as *μ*L of extract/1 mL of reaction volume.

(*6) Oxygen Radical Absorbance Capacity (ORAC)*. The oxygen radical absorbance capacity of BFE, BFD, and BFC was carried out according to the method of Ou* et al*. (2001) [20] with minor modifications in 96 well microplates. Phosphate buffer (75 mM, pH 7.4) was used to prepare 40 mg/mL 2, 2'-azobis (2-amidino-propane) dihydrochloride (AAPH), 4.8 *μ*M fluorescein, and 1.5 and 0.75 *μ*g/mL Trolox standard solutions. Initially, BFE, BFD, and BFC samples were dissolved in DMSO where the concentration of DMSO in samples was maintained less than 2%. A reaction volume of 200 *μ*L containing 4.8 *μ*M fluorescein and 50 *μ*L of samples (BFE: 3.91 *μ*L/mL; BFD: 3.91 *μ*L/mL and BFC: 62.5 *μ*L/mL) were preincubated at 37 °C for 10 minutes. Then reaction was initiated by adding 50 *μ*L of 40 mg/mL AAPH. The fluorescent microplate reader (SPECTRAmax- Gemini EM, Molecular Devices, USA) was set at 494 nm and 535 nm for excitation and emission. It was used to record the decay of fluorescein at 1 minute interval for 35 minutes. The net area under the curve of fluorescein decay, blank, and samples were compared to calculate the ORAC of the BFE, BFD, and BFC. Results were expressed as mg TE per litre of sample. Trolox was used as the standard.

#### 2.6.2. Evaluation of Antidiabetic Activity

(*1) Antiamylase Assay*. The alpha-amylase enzyme inhibitory activity of BFE, BFD, and BFC was determined according to the method of Bernfeld (1955) [21] with some modifications. Reaction volumes of 1 mL containing 40 *μ*L of starch (1% w/v), 50 *μ*L of alpha-amylase enzyme (5 *μ*g/mL) in sodium acetate buffer (100 mM, pH 6.0), and different concentrations of BFE, BFD, and BFC (BFE: 700, 350, 175 *μ*L, n = 3; BPD: 700, 350, 175 *μ*L, n = 3; BFC: 700, 350, 175 *μ*L, n = 3) were incubated at 40 °C for 15 minutes. After the incubation period, 500 *μ*L of DNS reagent was added and placed in a boiling water bath for 5 min and cooled using an ice water bath. The absorbance was measured at 540 nm using a microplate reader (SPECTRAmaxPLUS384, Molecular Devices, USA). For the control experiments, 100 mM sodium acetate buffer was used to replace identical BFE, BFD, BFC volumes while enzyme solutions were replaced by acetate buffer for the blank samples. Acarbose (6.25 – 100 *μ*g/mL, n = 4), a clinical inhibitor of alpha-amylase enzyme, was used as the positive control in this assay. Alpha-amylase inhibition was calculated using

(2) $$\begin{array}{c} Inhibition\;\;\left(\;\%\;\right)=\left[\;\frac{A_{c}-\left(A_{s}-A_{b}\right)}{A_{c}}\;\right]\times 100 \end{array}$$

where A_c_ is the absorbance of the control, A_b_ is the absorbance of sample blanks, and A_s_ is the absorbance in the presence of BFE, BFD, and BFC or acarbose.

(*2) Antiglucosidase Assay*. The alpha-glucosidase enzyme inhibitory activity of BFE, BFD, and BFC was evaluated according to the method described by Matsui* et al.* (2001) [22] in 96-well microplates with minor modifications. Reaction volumes of 100 *μ*L containing 35 *μ*L of different concentrations of BFE, BFD, and BFC [1000, 500, 250 *μ*L/mL in 50 mM sodium acetate buffer (pH 5.8); n=3], 50 *μ*L of 4 mM p-nitrophenyl-*α*-D-glucopyranoside (PNPG), and 35 *μ*L/mL of alpha-glucosidase enzyme were incubated at 37 °C for 30 minutes. After the incubation period, 50 *μ*L of 0.1 M Na_2_CO_3_ were added to the reaction mixtures and absorbance was measured at 405 nm using a microplate reader (SPECTRAmaxPLUS384, Molecular Devices, USA). Control experiment was carried out by providing similar conditions except replacing of samples by sodium acetate buffer while reaction volume without enzyme was used as the sample blank. Acarbose (0.25 – 2.50 *μ*g/mL, n = 3) was the positive control. Percentage inhibition of alpha-glucosidase enzyme was calculated using

(3) $$\begin{array}{c} Inhibition\;\;\left(\;\%\;\right)=\left[\;\frac{A_{c}-\left(A_{s}-A_{b}\right)}{A_{c}}\;\right]\times 100 \end{array}$$

where A_c_ is the absorbance of the control (100% enzyme activity), A_b_ is the absorbance produced by sample blanks, and A_s_ is the absorbance of the sample in the presence of BFE/ BFD/BFC/acarbose.

### 2.7. Analysis of Quality Parameters of Developed Blue Pea Flower Extract Incorporated Functional Beverage

As quality parameters pH, total soluble solids (TSS), titratable acidity (TA), colour and total plate count were estimated. All experiments were performed for both beverage samples with and without KMS. pH of blue pea flower extract incorporated beverage was measured by a digital pH meter (LPV2500.97.0002, sensION™+ PH 1, Spain) at 25 °C according to the AOAC 981.12 method [23]. Total soluble solid content (TSS) of the beverage was estimated using a handheld refractometer (Kyowa, HR-1, Japan) according to the method of AOAC 932.12 [23]. Titratable acidity (TA) was determined using AOAC 942.15 method [23]. Surface colour of the beverage was determined using a chroma meter (Konica Minolta INC-brand, CR-400, Japan) using 50 mL of the beverage and expressed against the scale of L*∗* (lightness), a*∗* (redness), b*∗* (yellowness) in the CIE (Commission Internationale de l'Eclairage) Lab system. Total plate count of the beverage was determined according to the method of SLS 516 Part 1: 2013 [24].

### 2.8. Evaluation of the Storage Stability of Blue Pea Flower Extract Incorporated Functional Beverage

Storage stability of the blue pea flower extract incorporated functional beverage with and without KMS was evaluated using TSS, TA, colour, pH, and microbial quality at different time intervals (1st, 14th, and 28th day of storage at room temperature).

### 2.9. Development of a Colour Chart for the Blue Pea Flower Extract Incorporated Functional Beverage

A colour chart was developed for blue pea flower extract incorporated functional beverage (BFD) with 14 different pH values ranging from 2 to 4 by adjusting the pH of BFD (pH values- 2.06, 2.14, 2.27, 2.33, 2.53, 2.65, 2.77, 2.86, 3.08, 3.12, 3.24, 3.54, 3.75, 3.98, n=5 each). Colour of each sample was measured using a chroma meter (Konica Minolta INC-brand, CR-400, Japan) and expressed in terms of L*∗* (lightness), a*∗* (redness), b*∗* (yellowness) in the CIE (Commission Internationale de l'Eclairage) Lab system.

### 2.10. Statistical Analysis

Statistical analysis of the Box–Behnken design was done using analysis of variance (ANOVA) to identify the significance of the model and independent variables using the Design Expert Software (Version 10.0.0, Stat-Ease Inc, Minneapolis, MN, USA). Verification of the model was done by comparing the predicted value of the model and the real value obtained following the optimized conditions by a t-test using Minitab software (Version 15.1.0, Minitab, Inc, Pennsylvania, USA).

Results of the ranking test were analysed by Friedman test and mean separation was done by the Wilcoxon sign rank test. Median of the 9-point hedonic test was tested using the Wilcoxon sign rank test.

Data of each other experiment were statistically analysed. One way analysis of variance (ANOVA) and the Duncan's Multiple Range Test were used to determine the differences among treatments at the significance level of 0.05. All the statistical analyses were conducted using SPSS software (Version 20.0) and performed in triplicate and the results were presented as mean values with standard deviation (±SD).

## 3. Results and Discussion

### 3.1. Optimization of Extraction Procedure of Blue Pea Flower

Results obtained for 15 treatment combinations in Box–Behnken model are given in Table 2. In this study, the TPC values ranged from 23.84±4.05 to 81.12±4.65 mg GAE/L of extract (18.62 to 27.84 mg GAE/g of flower) for different conditions used. Fitting the model for all linear and quadratic terms of independent variables were done by regression analysis and a multiple regression equation was obtained to predict the yield of total phenolic content as follows:

(4) TPC=51.59−1.77A−0.59B+23.99C−3.45AB−0.15AC−6.20BC−1.48A2−6.25B2+0.96C2

where TPC is the total polyphenolic content (mg GAE/L of extract), and A, B, C are independent variables such as temperature (°C), time (min), and F: W ratio, respectively.

**Figure 2:**
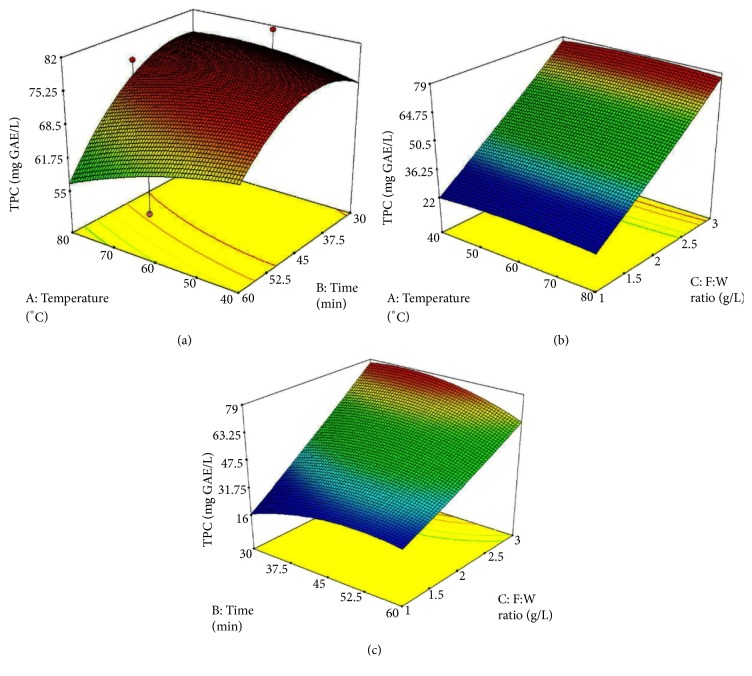
Response surface graphs for the interaction effects of temperature, time, and F: W Ratio (g/L) on extraction yield of TPC; (a) TPC versus temperature and time, (b) TPC versus temperature and F: W ratio, (c) TPC versus time and F: W ratio.

Regression correlation (R^2^) value between TPC and extraction conditions was 0.93 which represents an acceptable correlation. Results of the analysis of variance indicated that only F: W ratio had a significant effect (p<0.05) on the extraction of TPC whereas extraction temperature and extraction time and all the interaction effects were not significant (p>0.05). The predicted model was significant (p<0.05) and lack of fit of the test was not significant (p>0.05) which indicated that the fitted model was sufficient for prediction within the design space. Further, three-dimensional response surface plots (Figure 2) were used to illustrate the relationship between independent variables and the response variable where one variable was kept constant at the center point of the testing range and other two factors within the experimental range were depicted in 3-dimensional surface plots.

The optimum extraction conditions predicted using desirability function method were 59.6 °C of extraction temperature, 37 minutes of extraction time, 3 g/L of F: W ratio and the maximum TPC value expected was 78.38 mg GAE/L of extract. The experimental TPC obtained following the predicted optimized extract conditions (80.17±6.51 GAE/L of extract) was not significantly different (p>0.05) from the predicted value.

### 3.2. Development of Blue Pea Flower Extract Incorporated Functional Beverage

**Figure 3:**
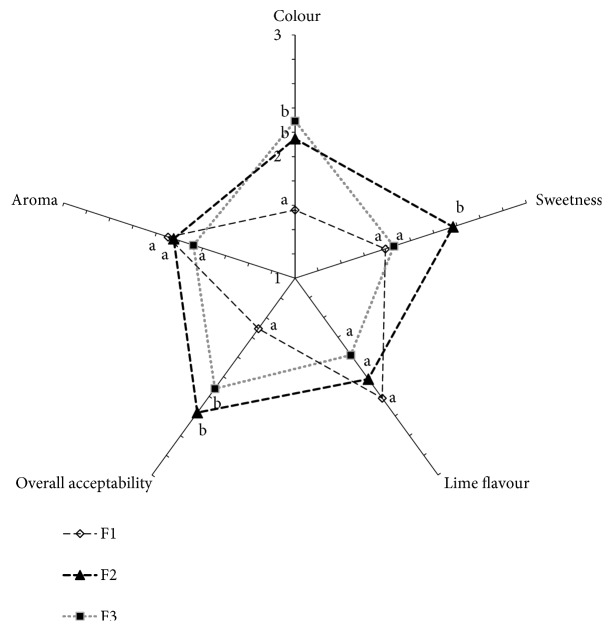
Sensory radar chart of sensory attributes of different formulations in ranking test. (^a, b^Mean values (n=41) in a scale are significantly different at p<0.05).

Out of 9 formulations developed in preliminary studies, 3 most acceptable formulations (F1, F2, and F3) in terms of tested sensory attributes (colour, aroma, lime flavour, sweetness, and overall acceptability) were selected and given in Table 3. Results of the ranking test conducted for selected three formulations (F1, F2, and F3) are given in Figure 3. According to the statistical analysis, formulations were not significantly different (p>0.05) for the preference of lime flavour and aroma. However, the preference for colour, sweetness, and overall acceptability of 3 beverage samples was significantly different (p<0.05). Further, the preference for F1 formulation was significantly higher (p<0.05) than the other two formulations with respect to colour and overall acceptability. Meanwhile, F2 had the least preference for the sweetness while the preference for sweetness of F1 and F3 was not significantly different (p>0.05). Thus, F1 formulation was selected as the most acceptable formulation among F1, F2, and F3 formulations.

**Figure 4:**
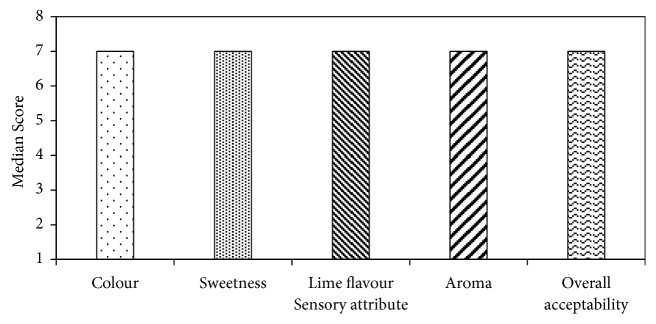
Median scores of consumer preference of the sensory attributes of selected best formulation (F_1_). (Median scores: 1- dislike extremely, 2- dislike very much, 3- dislike moderately, 4- dislike slightly, 5- neither like nor dislike, 6- like slightly, 7- like moderately, 8- like very much, 9- like extremely.)

It (F1) was further evaluated using a 9-point hedonic scale and it obtained median scores (Figure 4) of 7 for all sensory attributes indicating a moderate likeness. Statistical analysis conducted using Wilcoxon signed rank test for medians showed that estimated median values of all attributes were significantly higher (p<0.05) than the median value of 5 in the 9-point hedonic scale.

### 3.3. Antioxidant Activity of the Blue Pea Flower Extract and Blue Pea Flower Extract Incorporated Functional Beverage

**Figure 5:**
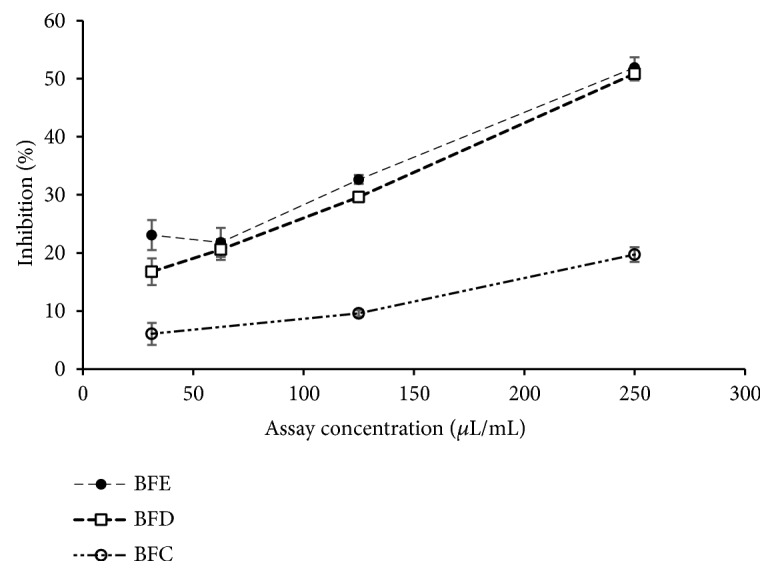
Dose response relationship of blue pea flower extract (BFE), blue pea flower extract incorporated functional beverage (BFD), and control experiment replacing blue pea flower extract with distilled water (BFC) for DPPH radical scavenging activity.

**Figure 6:**
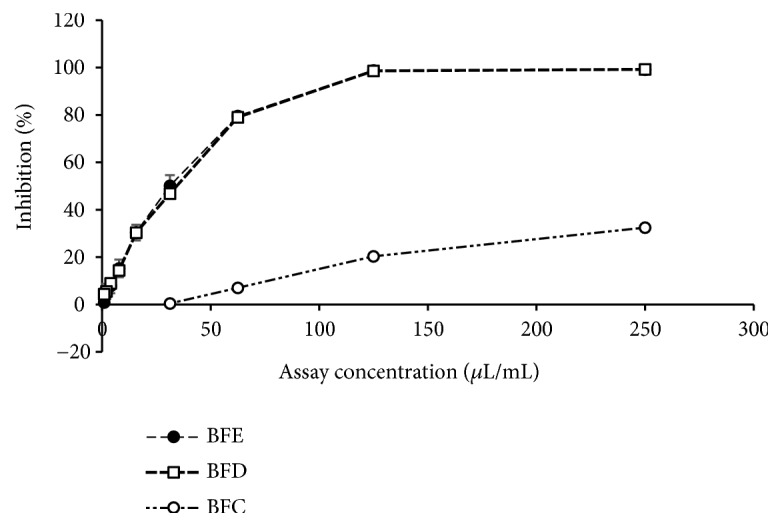
Dose response relationship of blue pea flower extract (BFE), blue pea flower extract incorporated functional beverage (BFD), and control experiment replacing blue pea flower extract with distilled water (BFC) for ABTS radical scavenging activity.

BFE, BFD, and BFC showed antioxidant activity with varying potential and results are given in Tables 4 and 5. Total phenolic content of BFE, BFD, and BFC ranged from 10.75±1.42 to 85.57±4.18 mg GAE/L. Total flavonoid content of BFE, BFD, and BFC ranged from 1.96±0.22 to 43.67±2.30 mg QE/L. DPPH radical scavenging activity of BFE, BFD, and BFC ranged from 11.51±0.32 to 35.92±1.15 mg TE/L while IC_50_ values varied from 241.84±7.84 to 754.41±20.95 *μ*L/ mL. ABTS^+^ radical scavenging activity of BFE, BFD, and BFC ranged from 18.48±0.41 to 192.14±9.75 mg TE/L where IC_50_ values varied from 34.71±1.80 to 360.32±8.05 *μ*L/mL. The dose response relationships of BFE, BFD, and BFC for DPPH and ABTS radical scavenging activities are given in Figures 5 and 6, respectively. Oxygen radical absorbance capacity of BFE, BFD, and BFC varied from 10.26±3.11 to 122.28±7.26 mg TE/L. In addition, FRAP of BFE, BFD, and BFC ranged from 4.17±0.85 to 15.39±1.63 mg TE/L.

Blue pea flower extract enriched functional beverage had a significantly higher (p<0.05) antioxidant potential compared to both BFE and BFC in terms of TPC and ORAC. However, FRAP, TFC, DPPH, and ABTS of BFD were not significantly different (p>0.05) to BFE while BFC had the lowest antioxidant potential with respect to the tested experiments.

The higher level of TPC and ORAC in BFD than BFE could be due to the contribution of phenolic compounds and oxygen radical absorbance capacity from lime juice which were indicated by the considerable amount of antioxidants in BFC. Hence, the beverage also contained considerably a higher TPC and ORAC than the extract. In another study, TPC value for aqueous BFE which was extracted under room temperature for 1 hour with deionized water was reported as 20.7 mg GAE/g [25] which indicated that TPC obtained for BFE (26.72±2.17 mg GAE/g of dry flower = 80.17±6.51 mg GAE/L) in this study was comparatively high.

The contribution to FRAP, ABTS, and DPPH from lime juice was at a considerable level. It was evident that lime juice can compensate for the loss of antioxidant activity from the extract due to thermal degradation during pasteurization. Hence, the product contained a similar amount of FRAP, ABTS, and DPPH compared to the extract.

### 3.4. Antidiabetic Activity of the Blue Pea Flower Extract and Blue Pea Flower Extract Incorporated Functional Beverage

Antiamylase and antiglucosidase activities of BFE, BFD, and BFC are given in Table 6. BFE, BFD, and BFC did not demonstrate alpha-glucosidase inhibitory activity. Further, only BFE showed a mild antiamylase activity (4.28±1.02 % inhibition at 700 *μ*L/mL concentration) compared to the reference drug acarbose (IC_50_ 133.88±2.54 *μ*g/mL).

Daisy* et al*. (2009) [26] has reported the antihyperglycaemic effect of blue pea flower extract on alloxan induced diabetic rats. Further, several research studies have clearly shown that oxidative stress plays a key role in pathological processes observed in diabetes mellitus. The use of antioxidant therapy has shown beneficial effects for the management of pathologies associated with oxidative stress in diabetes patients [27]. For an example, a recent clinical study carried out by Chusak* et al.* (2018) [6] has reported that there is a positive effect from a beverage developed from* Clitoria ternatea* L. flower on glycaemic regulatory properties and antioxidant properties via different mechanisms in human subjects. However, the variety of flower used in that study was not mentioned which is very important as there are sufficient evidences mentioning that blue and white flower varieties of* Clitoria ternatea* L. have different levels of functional properties [5]. However, observed antidiabetic activity in the present study and previous studies may be at least partly due to the antioxidant properties of blue pea flower extract.

### 3.5. Quality Parameters and Storage Stability of Blue Pea Flower Extract Incorporated Functional Beverage

Physiochemical and microbial quality parameters of freshly prepared beverage samples with and without KMS were summarized in Table 7 (Day 1 parameters). According to the statistical analysis, pH, titratable acidity, TSS, total plate count, and colour (L*∗* and a*∗* value) of two samples were not significantly different (p>0.05). However, b*∗* value of samples was significantly different (p<0.05). This may be due to the bleaching of anthocyanins by added KMS [28]. Lower L*∗* values of beverage samples indicated a low lightness in samples while a*∗* values of beverage samples indicated that samples were red in colour than green in colour [29].

Titratable acidity and TSS of samples were much lower than the SLS 729:2010 standards for ready-to-serve fruit beverages [30] (maximum levels for titratable acidity and TSS are 1.0 % and 16%, respectively). Total plate counts of the beverage samples were very low compared to the SLS 729:2010 standard for ready-to-serve fruit drinks [30] (maximum value: 50 CFU/mL) which indicated a good microbial quality of the product. It may be due to the destruction of microorganisms in the product as a result of the pasteurization process and the antimicrobial properties of* Clitoria ternatea* L. flower extract which inhibit or destroy microorganisms in the product [31].

Storage stability (pH, TA, TSS, colour, and total plate count) of blue pea flower extract incorporated functional beverage with and without KMS was evaluated for the period of 28 days at different time intervals (1st, 14th, and 28th days) and results were given in Table 7. Storage studies indicated that all tested parameters (except b*∗* value) of blue pea flower extract incorporated beverage with and without KMS did not change significantly (p>0.05) during the storage period.

There was no microbial growth for the period of 14 days in both beverage samples with and without KMS. However, slight microbial growth was observed in both beverage samples in the 28th day of storage period although the level of the microbial count is much lesser than the maximum allowable level. The observed results may be due to the antimicrobial properties of flower extract of* Clitoria ternatea* L. [31] and the reduction of the initial microbial count by pasteurization of the beverage. Further, there was no significant difference (p>0.05) among blue pea flower extract incorporated functional beverage with and without preservatives for the period of 28 days of storage. Therefore, blue pea flower extract incorporated functional beverage without added preservatives is shelf stable for a period of 28 days.

### 3.6. Developed Colour Chart for the Blue Pea Flower Extract Incorporated Functional Beverage

**Figure 7:**
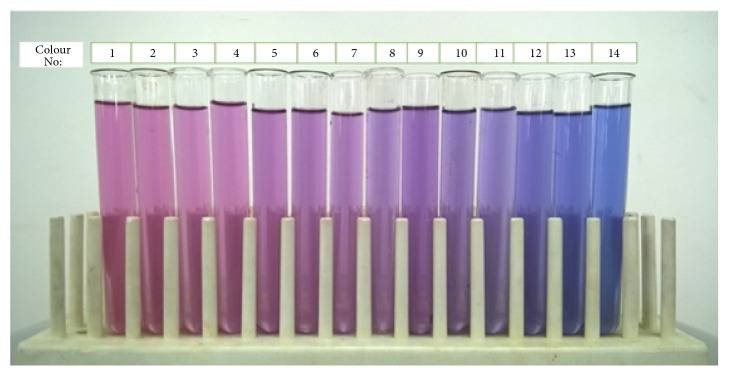
Developed colour chart for the blue pea flower extract incorporated functional beverage with 14 colours.

The colour values (L*∗*, a*∗*, b*∗*) of the product at 14 different pH values ranging from pH 2 to 4 were given in Table 8 and the developed colour chart with a corresponding colour number is given in Figure 7. It was observed that the L*∗* value (lightness), a*∗* value (redness), and b*∗* value (yellowness) decreased with increasing pH (Table 8). Beverages are susceptible for the pH changes due to the production of different acids such as lactic acid, acetic acid, formic acid, gluconic acid, and ethyl acetate by spoilage microorganisms [32]. In here, colour of the blue pea flower extract incorporated beverage changed due to the presence of anthocyanins in the* Clitoria ternatea* L. extract which is sensitive to pH [33]. Therefore, it can be used as a natural indicator which changes its colour with pH. In addition, colour characteristics of a product is an important parameter which influences the consumer acceptance of a product [34]. Hence, the developed colour chart can be used to monitor the quality and shelf-life of the product (Figure 7).

## 4. Conclusions

It is concluded that the optimum conditions for extraction of blue pea flower using RSM were 3 g of powdered blue pea flower/L of distilled water at 59.6 °C for 37 min and the most acceptable formulation consists of BFE, Stevia extract, and lime at a ratio of 983.25:1.75:15. Further, the developed blue pea flower extract incorporated beverage was shelf stable for a period of 28 days without preservatives. Therefore, the beverage is 100% natural and could be a better alternative for synthetic beverages. Since it possesses antioxidant properties via multiple mechanisms, it could be used as a functional natural beverage to manage oxidative stress associated with chronic diseases. However, glycaemic regulatory properties of the beverage via antiamylase and antiglucosidase activities were not detected in this study. Moreover, the colour chart developed can be used to monitor the quality of the product.

## Figures and Tables

**Table 1:** Three-factor, three-level Box-Behnken design used for response surface methodology (RSM) and experimental data for the RSM.

Treatment combinations^a^	Factor 1 (A) Temperature (°C)	Factor 2 (B) Time (min)	Factor 3 (C) F:W (g/L)
1	40 (-1)	45 (0)	1 (-1)
2	40 (-1)	60 (+1)	2 (0)
3	80 (+1)	60 (+1)	2 (0)
4	80 (+1)	45 (0)	3 (+1)
5	80 (+1)	45 (0)	1 (-1)
6	40 (-1)	30 (-1)	2 (0)
7	80 (+1)	30 (-1)	2 (0)
8	60 (0)	60 (+1)	1 (-1)
9	40 (-1)	45 (0)	3 (+1)
10	60 (0)	30 (-1)	3 (+1)
11*∗*	60 (0)	45 (0)	2 (0)
12*∗*	60 (0)	45 (0)	2 (0)
13	60(0)	30 (-1)	1 (-1)
14	60 (0)	60 (+1)	3 (+1)
15*∗*	60 (0)	45 (0)	2 (0)

^a^ Randomized.

*∗*Centre points.

**Table 2:** Experimental data of three-factor, three-level Box-Behnken design for the Response Surface Methodology.

Treatment combination^a^	Response TPC (mg GAE/L of extract)^b^
1	25.60±1.40
2	55.67±4.59
3	42.56±2.66
4	76.26±5.47
5	25.03±3.15
6	38.25±4.49
7	38.96±3.31
8	23.84±4.05
9	77.42±3.28
10	81.16±4.65
11	55.22±2.35
12	55.68±5.84
13	24.33±2.36
14	55.87±2.85
15	43.86±5.84

^a^Randomized, ^b^Mean± SD of triplicates.

**Table 3:** Selected formulations from preliminary trials.

Formulation (F)	Lime Juice (g/L)	Stevia (mL/L)	Flower Extract (mL/L )
F1	15	1.75	983.25
F2	20	1.50	978.50
F3	20	2.00	978.00

**Table 4:** Total phenolic content, total flavonoid content, ferric reducing antioxidant power, and oxygen radical absorbance capacity of blue pea flower extract (BFE), blue pea flower extract incorporated functional beverage (BFD), and control experiment replacing blue pea flower extract with distilled water (BFC).

Sample	TPC (mg GAE/L of sample)	TFC (mg QE/L of sample)	FRAP (mg TE/L of sample)	ORAC (mg TE/L of sample)
BFE	80.17±6.51^b^	42.75±1.74^a^	15.39±1.63^a^	110.18±3.19^b^
BFD	85.57±4.18^a^	43.67±2.30^a^	14.99±3.43^a^	122.28±7.26^a^
BFC	10.75 ±1.42^c^	1.96 ±0.22^b^	4.17±0.85^b^	10.26±3.11^c^

^a, b, c^Mean values (n=3) in a column are significantly different at p<0.05.

**Table 5:** DPPH radical scavenging activity and ABTS^+^ radical scavenging activity of blue pea flower extract (BFE), blue pea flower extract incorporated functional beverage (BFD), and control experiment replacing blue pea flower extract with distilled water (BFC).

Sample	DPPH radical scavenging activity		ABTS^+^ radical scavenging activity	
	mg TE/L	IC_50_ *μ*L/mL	mg TE/L	IC_50_ *μ*L/mL
BFE	35.92±1.15^a^	241.84±7.84^b^	192.14±9.75^a^	34.71±1.80^b^
BFD	35.07±0.64^a^	247.61±4.54^b^	185.81±2.68^a^	35.83±0.52^b^
BFC	11.51±0.32^b^	754.41±20.95^a^	18.48±0.41^b^	360.32±8.05^a^

^a, b^Mean values (n=3) in a column are significantly different at p<0.05.

**Table 6:** Antiamylase and antiglucosidase activity of blue pea flower extract (BFE), blue pea flower extract incorporated functional beverage (BFD), and control experiment replacing blue pea flower extract with distilled water (BFC).

Sample	Inhibition level of alpha-amylase enzyme (%)*∗∗*	Inhibition level of alpha-glucosidase enzyme (%)*∗*
BFE	4.28±1.02	ND
BFD	ND	ND
BFC	ND	ND

*∗*Percentage inhibition at 116.67 *μ*L/ mL concentration.

*∗∗*Percentage inhibition at 700 *μ*L/ mL concentration.

ND: value represents no activity.

IC_50_ values for alpha-amylase and *α*- glucosidase inhibitory activities of acarbose: 133.88±2.54 and 0.47±0.01 *μ*g/ml, respectively.

**Table 7:** Physicochemical, microbial quality analysis and storage studies of the beverage.

Parameter		Units	Day 1		Day 14		Day 28	
			With KMS	Without KMS	With KMS	Without KMS	With KMS	Without KMS
pH (25 °C)		-	3.12±0.01^a^	3.12±0.01 ^a^	3.12±0.01^a^	3.13±0.01^a^	3.14±0.01^a^	3.14±0.01^a^
TA		%	0.13±0.00^a^	0.13±0.00 ^a^	0.13±0.00^a^	0.13±0.00^a^	0.13±0.00^a^	0.13±0.00^a^
TSS		%	1 ^a^	1 ^a^	1 ^a^	1 ^a^	1 ^a^	1 ^a^
Colour	L*∗*	-	3.44±0.4 ^a^	3.72±0.07 ^a^	2.95±0.69^a^	3.88±0.18^a^	3.43±0.29^a^	3.87±0.27^a^
	a*∗*	-	4.83±0.09 ^a^	4.69±0.09 ^a^	4.93±0.11^a^	4.79±0.12^a^	4.89±0.17^a^	4.88±0.11^a^
	b*∗*	-	-0.06±0.05 ^a^	0.4±0.05 ^b^	-0.06±0.08^a^	0.36±0.06^b^	-0.03±0.11^a^	0.24±0.08^b^
Total plate count		CFU/mL	Nil	Nil	Nil	Nil	1.5±0.71^a^	2.5±0.71^a^

Values are expressed as mean±SD. ^a, b^Mean values in a row were significantly different (p<0.05). L*∗*: lightness, a*∗*: redness, b*∗*: yellowness, KMS: potassium metabisulphite, TA: titratable acidity, TSS: total soluble solids.

**Table 8:** L*∗*, a*∗*, and b*∗* values of the product at different pH values.

Colour No.	pH	L*∗*	a*∗*	b*∗*
1	2.06	4.02±0.03	8.04±0.20	3.19±0.13
2	2.14	4.59±0.06	7.62±0.11	2.89±0.1
3	2.27	4.36±0.15	6.97±0.10	2.47±0.11
4	2.33	4.50±0.33	6.92±0.16	2.01±0.07
5	2.53	4.21±0.30	5.81±0.10	1.75±0.05
6	2.65	4.18±0.11	5.43±0.12	1.55±0.18
7	2.77	3.31±0.07	5.36±0.06	1.04±0.09
8	2.86	4.06±0.12	5.01±0.12	0.86±0.13
9	3.08	3.90±0.07	4.11±0.15	1.56±0.2
10	3.12	3.72±0.07	4.78±0.07	0.27±0.07
11	3.24	1.95±0.25	4.58±0.11	0.06±0.11
12	3.54	1.66±0.30	4.33±0.08	-0.41±0.07
13	3.75	1.84±0.23	4.39±0.04	-0.93±0.11
14	3.98	1.92±0.22	4.47±0.07	-1.2±0.07

## Data Availability

The data used to support the findings of this study are available from the corresponding author upon request

## Supplementary Materials

Supplementary Materials Beverage formulations studied during preliminary studies.
