# Supplementary material for: A Commercial Potential Blue Pea (Clitoria ternatea L.) Flower Extract Incorporated Beverage Having Functional Properties
Source: Evid Based Complement Alternat Med. 2019 May 20;2019:2916914. doi: 10.1155/2019/2916914 (PMC6545798; doi:10.1155/2019/2916914)
Supplement: Supplementary Materials — Beverage formulations studied during preliminary studies. [file 2916914.f1.pdf]

Table 1: Formulations studied in preliminary trials

Formulation (F)	Lime Juice (g/L)	Stevia (mL/L)	Flower Extract (mL/L)
F1*	15	1.75	983.25
F2*	20	1.50	978.50
F3*	20	2.00	978.00
F4	15	1.50	983.50
F5	15	2.00	983.00
F6	20	1.75	978.25
F7	25	1.50	973.50
F8	25	1.75	973.25
F9	25	2.00	973.00

*Selected formulations
